# Prime-boost vaccination with recombinant protein and adenovirus-vector expressing *Plasmodium vivax* circumsporozoite protein (CSP) partially protects mice against Pb/Pv sporozoite challenge

**DOI:** 10.1038/s41598-017-19063-6

**Published:** 2018-01-18

**Authors:** Tarsila Mendes de Camargo, Elisângela Oliveira de Freitas, Alba Marina Gimenez, Luciana Chagas Lima, Karina de Almeida Caramico, Kátia Sanches Françoso, Oscar Bruna-Romero, Chiara Andolina, François Nosten, Laurent Rénia, Hildegund C. J. Ertl, Ruth S. Nussenzweig, Victor Nussenzweig, Mauricio M. Rodrigues, Arturo Reyes-Sandoval, Irene S. Soares

**Affiliations:** 1 Department of Clinical and Toxicological Analyses, School of Pharmaceutical Sciences, University of São Paulo, São Paulo, SP Brazil; 2 The Jenner Institute, Nuffield Department of Medicine, University of Oxford, The Henry Wellcome Building for Molecular Physiology, Roosevelt Drive, Oxford, UK; 3 Center of Cellular and Molecular Therapy, Department of Microbiology, Immunology and Parasitology, Federal University of São Paulo, São Paulo, SP Brazil; 4 Department of Microbiology and Immunology, Federal University of Santa Catarina, Florianópolis, SC Brazil; 5 Shoklo Malaria Research Unit, Mahidol-Oxford Tropical Medicine Research Unit, Faculty of Tropical Medicine, Mahidol University, Mae Sot, Thailand; 6 Centre for Tropical Medicine and Global Health, Nuffield Department of Medicine Research building, University of Oxford Old Road campus, Oxford, UK; 7 Singapore Immunology Network, Biopolis, Agency for Science Technology and Research, Singapore, Singapore; 8 The Wistar Institute, Philadelphia, PA USA; 9 Michael Heidelberger Division, Department of Pathology, New York University School of Medicine, New York, NY USA

## Abstract

Vaccine development against *Plasmodium vivax* malaria lags behind that for *Plasmodium falciparum*. To narrow this gap, we administered recombinant antigens based on *P. vivax* circumsporozoite protein (CSP) to mice. We expressed in *Pichia pastoris* two chimeric proteins by merging the three central repeat regions of different CSP alleles (VK210, VK247, and *P. vivax*-like). The first construct (yPvCSP-All_FL_) contained the fused repeat regions flanked by N- and C-terminal regions. The second construct (yPvCSP-All_CT_) contained the fused repeat regions and the C-terminal domain, plus RI region. Mice were vaccinated with three doses of yPvCSP in adjuvants Poly (I:C) or Montanide ISA720. We also used replication-defective adenovirus vectors expressing CSP of human serotype 5 (AdHu5) and chimpanzee serotype 68 (AdC68) for priming mice which were subsequently boosted twice with yPvCSP proteins in Poly (I:C) adjuvant. Regardless of the regime used, immunized mice generated high IgG titres specific to all CSP alleles. After challenge with *P. berghei* ANKA transgenic parasites expressing Pb/PvVK210 or Pb/PvVK247 sporozoites, significant time delays for parasitemia were observed in all vaccinated mice. These vaccine formulations should be clinically tried for their potential as protective universal vaccine against *P. vivax* malaria.

## Introduction

Malaria is one of the most prevalent infectious diseases. It is caused by *Plasmodium* parasites that are transmitted through the bite of *Anopheles* mosquitoes in tropical regions. The discovery of alternative tools to control malaria is increasingly important because of the emergence of drug-resistant parasites and insecticide-resistant mosquitoes. In 2015, 214 million malaria cases and over 438,000 deaths occurred globally, indicating that the disease remains a significant public health concern^1^. In humans, malaria is caused by five species: *Plasmodium falciparum, P. vivax, P. malariae*, *P. ovale*, *and P. knowlesi*. Among the *Plasmodium* species, *P. vivax* is the most widely distributed worldwide and is most prevalent in the American continent. An increase in the number of cases with serious complications has been reported recently in several countries^2^; these complications include anaemia, respiratory distress syndrome, cerebral malaria, and malnutrition. In Brazil, 80% of malaria cases reported in 2015 (about 143,000 infected people) were caused by *P. vivax*^1^.

According to the malERA (Malaria Eradication Research Agenda) consultative group, vaccination should control and, one day, even eliminate malaria. There is no approved vaccine against malaria thus far; however, some formulations are currently under clinical testing^3^. A malaria vaccine presently in late-phase clinical testing, RTS,S, manufactured by GlaxoSmithKline (GSK), is based on the *P. falciparum* circumsporozoite protein (*Pf*CSP). RTS,S is a recombinant vaccine produced in *Saccharomyces cerevisiae*, which comprises the C-terminal portion of *Pf*CSP, and repeat regions fused to the surface antigen (S) of hepatitis B virus^4^. This vaccine may contribute substantially to the control of malaria when used in combination with other control measures, especially in high-risk areas^5^. However, this vaccine targets only *P. falciparum*, as do almost all vaccine candidates tested so far^3^.

In contrast with *Pf*CSP, *P. vivax* CSP has three allelic variants. This protein has B cell immunodominant epitopes consisting of repeats of core amino acids, and the two most common genotypic variants are VK210 and VK247^6–8^. Each variant displays two main types of repetitions of nonapeptides: GDRA(D/A)GQPA and ANGA(G/A)(C/D)QPG, respectively^8^. A third variant of a human malaria parasite called “*P. vivax*-like” containing the repeat APGANQ(E/G)GAA^9,10^ has been found in endemic regions of Papua New Guinea, as well as in Brazil, Indonesia, and Madagascar^10^. In Brazil, the prevalence of the three PvCSP allelic variants has been established^11,12^.

A subunit vaccine against *P. vivax* malaria is currently undergoing clinical testing. This vaccine, named VMP001, was expressed in *Escherichia coli* and encodes a chimeric CS protein containing repeat sequences of the two major alleles, VK210 and VK247^10^. Mice immunized with protein in Freund’s adjuvant produced antibodies (Abs) that recognize both VK210 and VK247 alleles and cause the agglutination of live sporozoites^13^. This protein was further tested in formulations with a Toll-like receptor (TLR)-9 agonist in *Aotus nancymaae* monkeys. Animals generated strong humoral immune responses, and protection was associated with Abs directed against the central regions of VK210 and VK247^14^. Results of the first Phase 1 trial with VMP001 showed that the vaccine was well tolerated and immunogenic, and all volunteers generated robust humoral and cellular responses to the vaccine antigen. Vaccination did not induce sterilizing protective immunity; however, a significant time delay for parasitemia was observed in 59% of vaccinated individuals compared to that seen in the control group^15^.

A strategy for improving the humoral response of protein-based subunit vaccines is to use adenoviral vectors in heterologous prime-boost immunization regimens. Adenoviral vectors efficaciously induced long-lasting humoral and cellular immune responses in different models including malaria^16–18^. Moreover, these responses were associated with protection against *Plasmodium* infection in several studies^16,19–22^. Over the past decade, our group has generated and evaluated the immunogenicity of recombinant proteins and adenoviruses representing the three forms of *P. vivax* CSP. Recombinant proteins were initially expressed in *Escherichia coli*^23–25^ and their immunogenicities were tested by immunizing C57BL/6 mice with each protein in combination with Freund’s adjuvants (CFA/IFA) or the agonist of Toll-like receptor 3, Polyinosinic-polycytidylic acid [Poly (I:C)]. All recombinant proteins were highly immunogenic when administered either individually or together and specific Abs against all three allelic variants were obtained with either adjuvant, Freund’s or Poly (I:C). The Abs produced were directed against the CSP immunodominant domain repeats, N- and C-terminal regions^25^.

In this work, we used two chimeric proteins expressed as soluble products in *Pichia pastoris*. These recombinants merged the RI region with the three central repeat regions of different CSP alleles (VK210, VK247, and *P. vivax*-like), and also contained N- and C-terminal (yPvCSP-All_FL_) or only C-terminal (yPvCSP-All_CT_) regions from *P. vivax* CSP. In addition, we also used two adenoviral vectors (AdHu5 or AdC68) in heterologous prime-boost immunization regimens, and subsequently challenged immunized mice with transgenic *P. vivax/P. berghei* sporozoites (Pb/Pv).

## Results

### Two chimeric proteins based on PvCS were successfully produced in yeast

**Figure 1:**
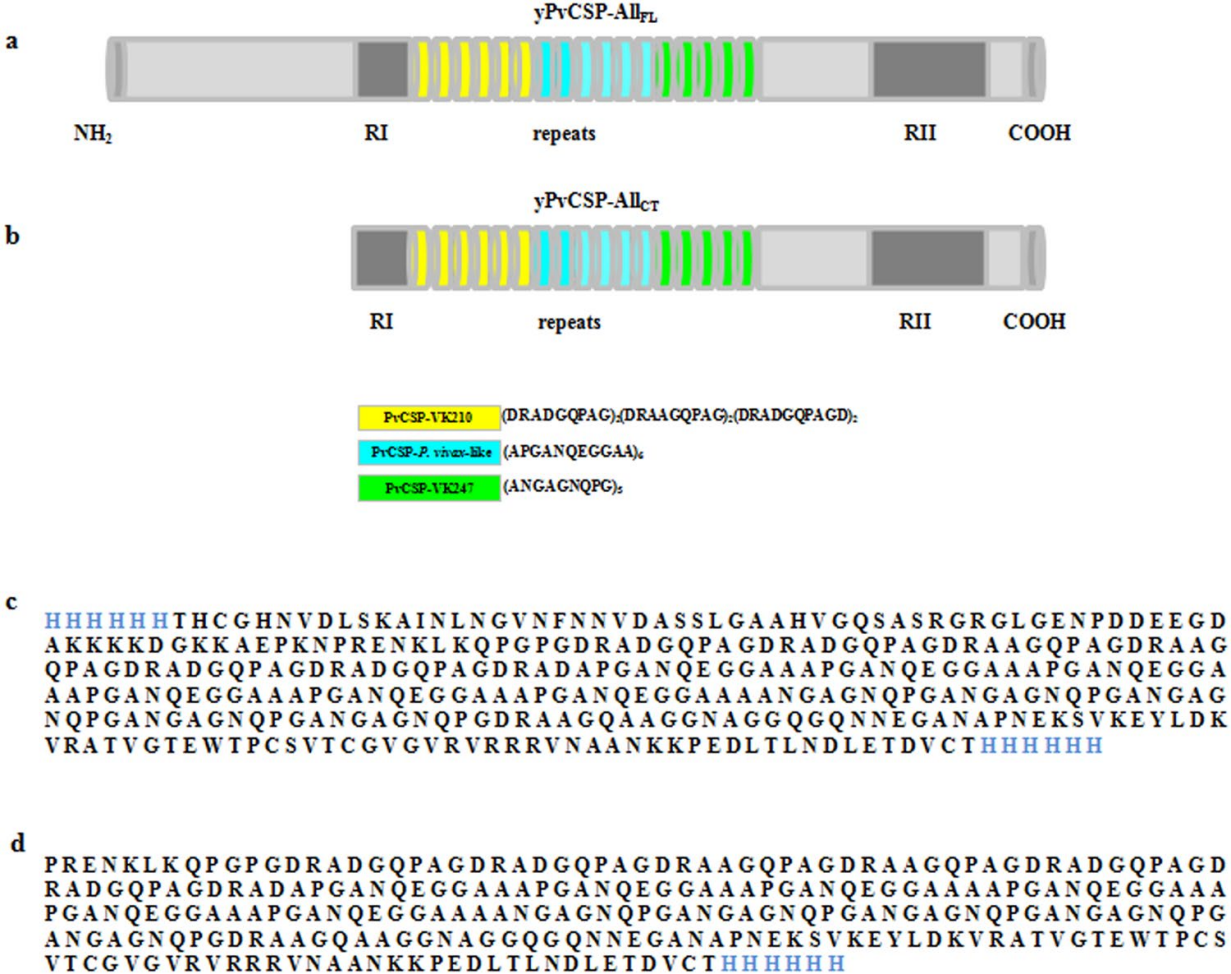
Representation of *Plasmodium vivax* circumsporozoite (CS) proteins expressed from *Pichia pastoris*. (**a**) Schematic representation of the construction and open reading frame of protein PvCSP-All_FL_. (**b**) PvCSP-All_CT_. Sequence repeats in the variant PvCSP-VK210 are indicated in yellow; PvCSP-*P. vivax*-like in blue; and PvCSP-VK247 in green. (**c**) Amino acid sequence of the protein yPvCSP-All_FL_. (**d**) Amino acid sequence of the protein yPvCSP-All_CT_. Hexa-histidine sequences are indicated in blue.

The final constructs of CSP used in this study have a repeat region that merges with the three variant repeat sequences reported until date: VK210, VK247, and *P*. *vivax*-like. The chimeric CSP in this study is composed of six repeats present in *P. vivax* VK210, six from *P. vivax*-like, and five from VK247. The structure of the final constructs of yPvCSP-All_FL_ and yPvCSP-All_CT_ are described in Fig. 1(a,b).

**Figure 2:**
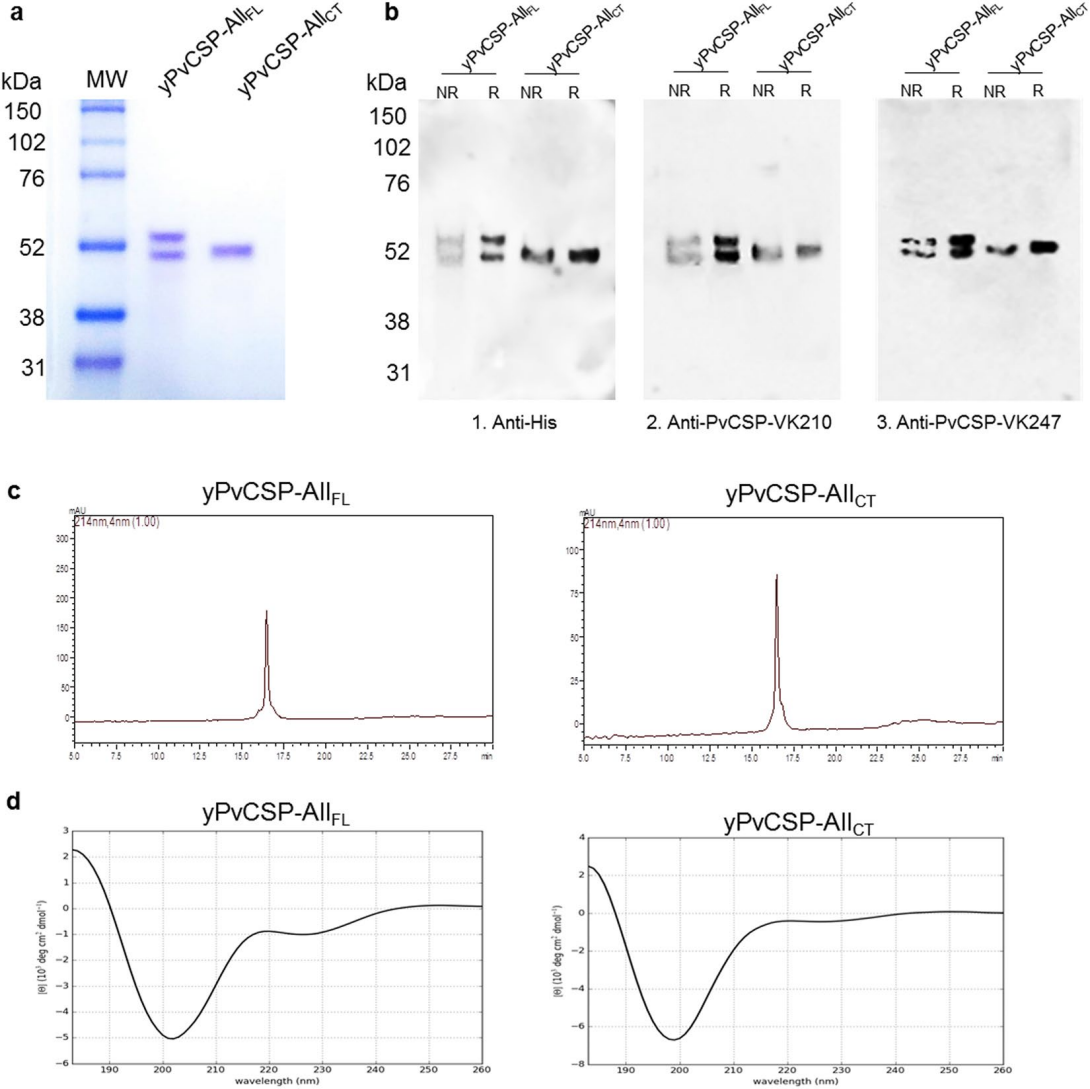
Expression, purification, and biophysical analyses of recombinant proteins. (**a**) SDS-PAGE (12%) of yPvCSP-All_FL_ and yPvCSP-All_CT_ proteins, stained with Coomassie blue under reducing conditions. (**b**) Immunoblotting analyses of recombinant yPvCSP-All_FL_ and yPvCSP-All_CT_. (1). Anti-histidine tag mAb (diluted 1:1,000, GE Healthcare); (2). Anti-PvCSP-VK210 mAb (diluted 1:1,000) 3. Anti-PvCSP-VK247 mAb (diluted 1:11,000). NR and R: proteins under non-reducing and reducing conditions (with 2-mercaptoethanol), respectively; (**c**) The purity of proteins yPvCSP-All_FL_ and yPvCSP-All_CT_, after a combination of chromatographic separations, was analysed by RP-HPLC. The gradient elution was developed with 0.1% TFA in water and 0.1% TFA in 90% acetonitrile at 22 °C, and a rate of 1 mL/min for 30 minutes on a C_18_ column (Phenomenex). MW: molecular weight. (**d**) The secondary structure of yPvCSP-All_FL_ and yPvCSP-All_CT_ was analysed by a circular dichroism (CD) spectrum. The CD spectrum of the recombinant proteins was recorded from 190 to 260 nm using a JASCO-J815 spectropolarimeter.

The protein yield was 3 mg/L of yPvCSP-All_FL_ and 8 mg/L of yPvCSP-All_CT_. Both proteins were purified as described in the Methods section by affinity chromatography and ion exchange chromatography, and the final purity was >95% for yPvCSP-All_FL_ and yPvCSP-All_CT_ according to RP-HPLC. Both proteins had molecular weights of ~55 kDa under reducing conditions (Fig. 2a).

The electrophoresis analysis showed that yPvCSP-All_FL_ and yPvCSP-All_CT_ migrated without degradation products, and the proteins were recognised by specific monoclonal Ab anti-PvVK210 and mAb anti-PvVK247^26^ (Fig. 2a,b). The results indicated that the introduction of limited copies of each PvCSP allelic variant was sufficient for recognition by specific mAbs. In addition, the two recombinant proteins were detected by mAb anti-His, indicating His_6_-tag preservation (Fig. 2b).

Reverse-phase chromatography on a C_18_ column, in which a single peak was observed, confirmed the high purity of the final yPvCSP-All_CT_ and yPvCSP-All_FL_ used in this study (Fig. 2c). To investigate the secondary structure, the recombinant proteins were examined by far-UV CD spectroscopy (Fig. 2d). The CD spectra were consistent with a structure comprising 2% α-helix, 43% β-sheet, and 55% β-turn for yPvCSP-All_CT_, and 3% α-helix, 41% β-sheet, and 56% β-turn for yPvCSP-All_FL_. The stability of the recombinant proteins following a freeze-drying process was also examined by SDS-PAGE (not shown). Both proteins preserved their conformation after the lyophilisation process.

### Mice immunized with yPvCSP-All_FL_ or yPvCSP-All_CT_ generated high IgG titres specific to three allelic forms of PvCSP

**Figure 3:**
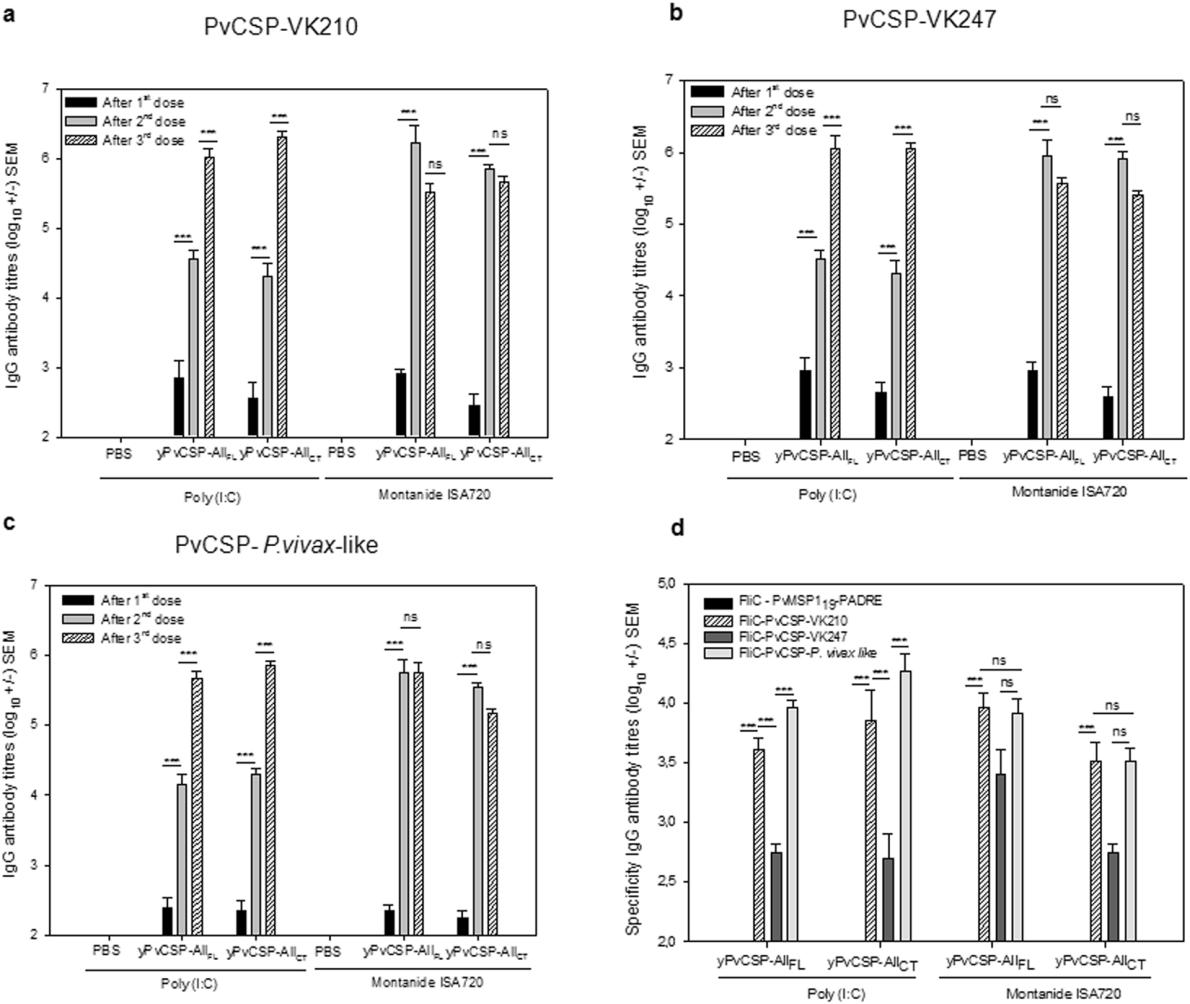
IgG Ab and specificity responses in immunized mice. Groups of 6- to 8-week-old female C57BL/6 mice (n = 6) were immunized subcutaneously with 10 µg of each recombinant protein and Poly (I:C) (50 µg/dose) or Montanide ISA720 (7:3). IgG titres were measured by ELISA against the homologous proteins and individual full-length PvCSP variants. (**a**) PvCSP-VK210. (**b**) PvCSP-VK247, and (**c**) PvCSP-*P. vivax*-like. The results are expressed as the arithmetic mean titres of each group (log_10_ ± SEM), and were compared by one-way ANOVA followed by Tukey test for multiple comparisons. Significant differences between groups are denoted on the graph as follows: *P < 0.05, **P < 0.01, and ***P < 0.001. Non-significant (ns) differences are indicated (P > 0.05). (**d**) The elicited Abs recognised the different allelic variants and were directed at the central repeats, as shown by assays against the repeats fused to flagellin FliC (FliC-PvCSP-repeats) after three doses.

C57BL/6 mice were immunized with 10 µg of each protein mixed with Poly (I:C) (50 µg/dose) adjuvant or emulsified in Montanide ISA720 (7:3). Regardless of which type of adjuvant was used, mice vaccinated individually with either yPvCSP-All_FL_ or yPvCSP-All_CT_ developed high IgG Ab titres (>10^5^) specific to all CSP alleles after three doses (PPP). No difference was observed in the IgG titres specific to CSP (Fig. 3) with the three CSP alleles, indicating that recognition was similar. After immunization with the three doses of yPvCSP-All_FL_/Montanide ISA720, mean IgG titres (log_10_ ± SEM) were 5.51 ± 0.12 to PvCSP-VK210, 5.56 ± 0.09 to PvCSP-VK247, and 5.70 ± 0.12 to PvCSP-*P. vivax-like*. The formulation yPvCSP-All_FL_/Poly (I:C) produced IgG titres of 6.01 ± 0.12 to PvCSP-VK210, 6.06 ± 0.17 to PvCSP-VK247, and 5.66 ± 0.12 to PvCSP-*P. vivax-like*.

In comparison, after three immunizations with yPvCSP-All_CT_/Poly (I:C), IgG titres (log_10_ ± SEM) were 6.13 ± 0.06 to PvCSP-VK210, 6.06 ± 0.06 to PvCSP-VK247, and 5.86 ± 0.05 to PvCSP-*P. vivax-like*. With the adjuvant Montanide plus yPvCSP-All_CT_, the IgG titres (log_10_ ± SEM) were 5.66 ± 0.09 to PvCSP-VK210, 5.42 ± 0.06 to PvCSP-VK247, and 5.16 ± 0.09 to PvCSP-*P. vivax-like*. IgG titres specific to CSP were significantly higher in animals vaccinated with Montanide ISA720 than in those that received Poly (I:C) after one boost (P < 0.001). However, both formulations were highly immunogenic after two boosts, with a potent humoral response elicited in C57BL/6 mice.

To determine the specificity of anti-yPvCSP-All_FL_ and anti-yPvCSP-All_CT_ Abs, mouse serum was tested for the three individual FliC-PvCSP-repeats. In addition, FliC-MSP1_19_-PADRE was used to exclude the possibility that Abs cross-react with the FliC region. By ELISA, significant differences were found in antibody titres to FliC-PvCSP-VK247 (P < 0.001) (Fig. 3d). After three doses of immunization with yPvCSP-All_FL_/Poly (I:C), mean IgG titres (log_10_ ± SEM) were 3.60 ± 0.10 to FliC-PvCSP-VK210, 2.75 ± 0.06 to FliC-PvCSP-VK247, and 3.96 ± 0.06 to FliC-PvCSP-*P. vivax*-like. The formulation yPvCSP-All_CT_/Poly (I:C) produced IgG titres of 3.85 ± 0.25 to FliC-PvCSP-VK210, 2.70 ± 0.20 to FliC-PvCSP-VK247, and 4.26 ± 0.15 to FliC-PvCSP-*P. vivax-like*. In comparison, after three immunizations with yPvCSP-All_FL_/Montanide ISA720, IgG titres were 3.96 ± 0.12 to FliC-PvCSP-VK210, 3.40 ± 0.20 to FliC-PvCSP-VK247, and 3.91 ± 0.12 to FliC-PvCSP-*P. vivax-like*. With the adjuvant Montanide plus yPvCSP-All_CT_, the IgG titres were 3.50 ± 0.15 to FliC-PvCSP-VK210, 2.75 ± 0.06 to FiC-PvCSP-VK247, and 3.50 ± 0.11 to FliC-PvCSP-*P. vivax-like*. As expected, animals vaccinated with the above formulations did not generate antibodies to FliC-MSP1_19_-PADRE. No significant differences were found in antibody titers specific to FliC-CSP in animals vaccinated with Poly (I:C) or Montanide ISA720.

**Figure 4:**
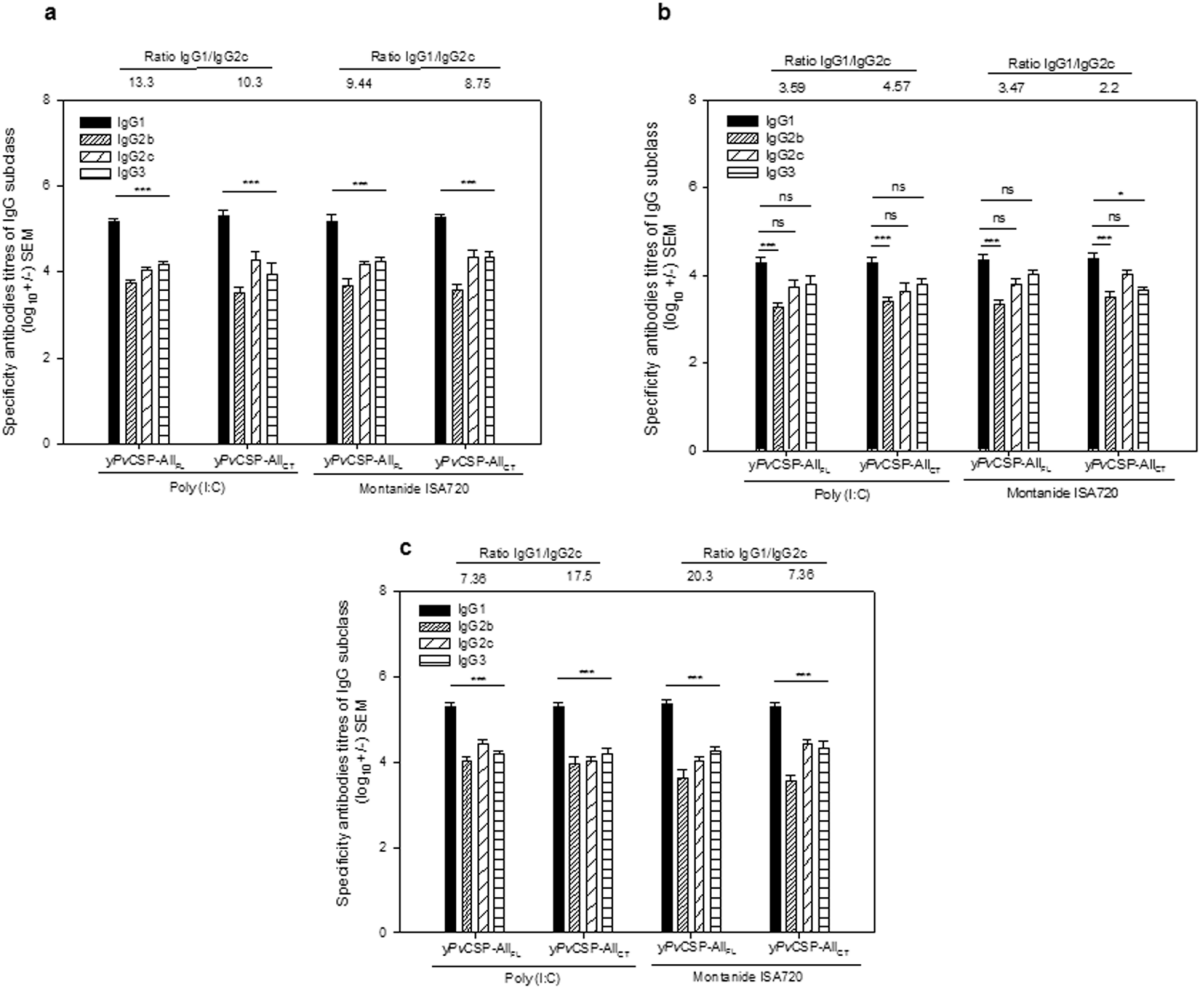
Ratio of IgG1/IgG2c to yPvCSP-All_FL_ and yPvCSP-All_CT_ after the immunization schedule. Chimeric proteins yPvCSP-All_FL_ and yPvCSP-All_CT_ induced Th2-polarised Abs in C57BL/6 mice. (**a**) FliC-PvCSP-VK210, (**b**) FliC-PvCSP-VK247, and **c**. FliC-PvCSP-*P. vivax*-like. The results are expressed as the arithmetic mean titres of each group (log_10_ ± SEM) and were compared by one-way ANOVA followed by Tukey test for multiple comparisons. Significant differences between groups are denoted on the graph as follows: *P < 0.05, **P < 0.01, and ***P < 0.001. Non-significant (ns) differences are also indicated (P > 0.05).

The subclasses of IgG Abs (IgG1, IgG2b, IgG2c, and IgG3) elicited against PvCSP-repeats were measured in sera after three doses (Fig. 4). High levels of IgG1 were observed in animals that received yPvCSP-All_FL_ or yPvCSP-All_CT_ with either adjuvant. A significant predominance of subclass IgG1 Abs (IgG1 > IgG2c, P < 0.001) elicited against allelic variants PvCSP-VK210 (Fig. 4a) and *P. vivax*-like (Fig. 4c) were determined. These results indicate a Th2-polarised response.

**Figure 5:**
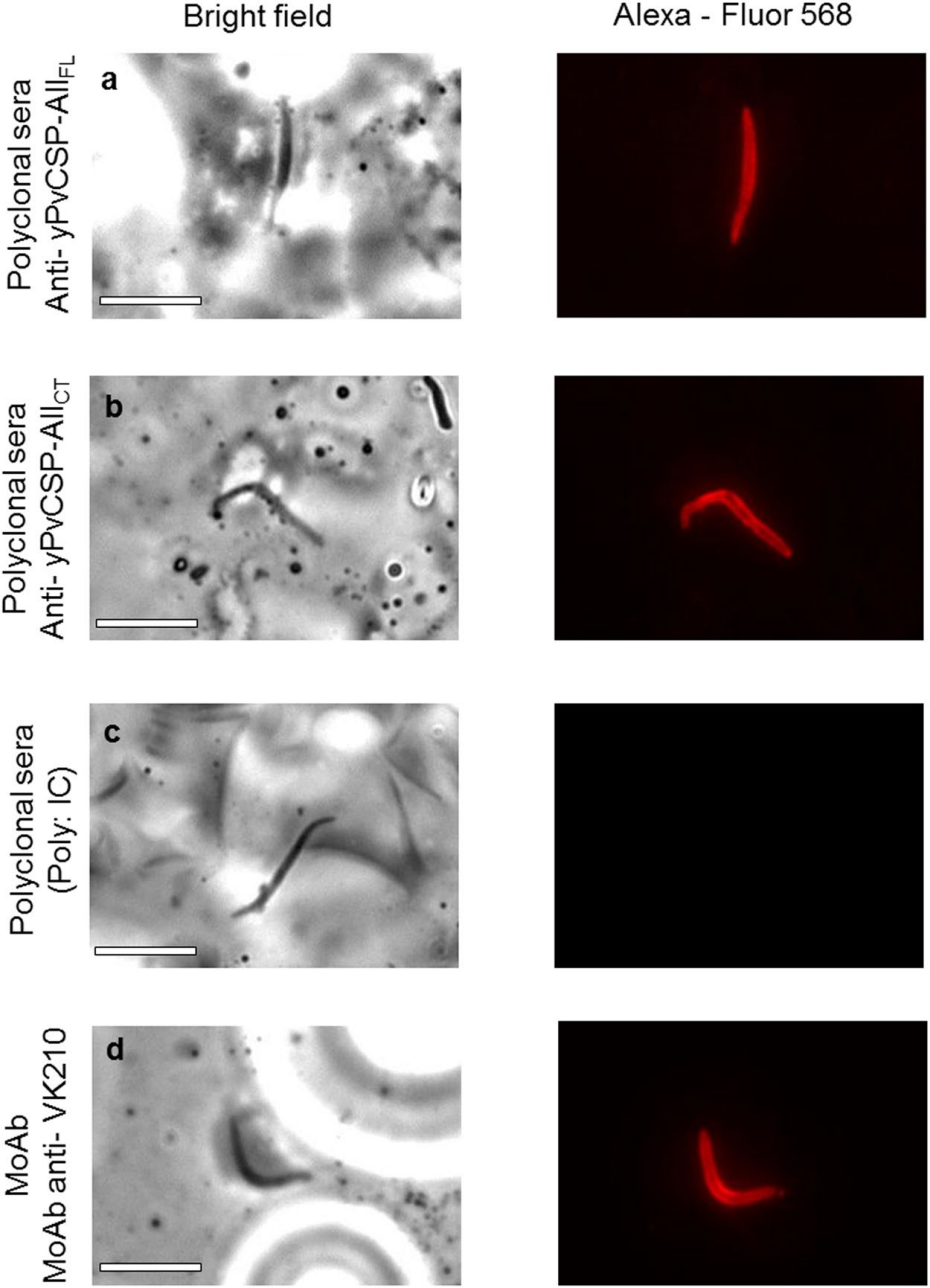
Indirect immunofluorescence analysis using sera from C57BL/6 mice. Microscope slides containing fixed spz of *P. vivax* obtained from patients from Thailand were incubated with pools of sera from mice immunized with yPvCSP-All in the presence of Poly (I:C). (**a**) Polyclonal sera anti-yPvCSP-All_FL_ (1:50). (**b**) Polyclonal sera anti-yPvCSP-All_CT_ (1:50). (**c**) sera from mice immunized with PBS in adjuvant (1:50). (**d**) mAb anti-VK210 (1:50). The white bars are equivalent to 10 micrometers (10 µm).

The affinity of Abs induced to chimeric yPvCSP-All_FL_ or yPvCSP-All_CT_ was tested by ELISA using bacterial constructions^25^ in the presence of a denaturing agent (6 M urea). Sera collected after the 3^rd^ dose with yPvCSP-All_FL_ or yPvCSP-All_CT_ and in the presence of adjuvants Poly (I:C) or Montanide ISA720 were incubated with PvCSP variants, in the presence and absence of 6 M urea. Avidity was calculated as percentage of OD_492_ obtained in presence of urea, considering 100% as the OD_492_ obtained in absence of urea^18^. The avidity of Abs induced in all vaccination protocols ranged between 40 and 60% to PvCSP-VK210, PvCSP-VK247 and PvCSP-*P. vivax*-like (Table 1). MAbs used as control (mAb anti-VK210 and mAb anti-VK247) had a higher avidity, 81.39% and 85.05%, induced against the respective inducing proteins PvCSP-VK210 and PvCSP-VK247, demonstrating a strong antigen-Ab affinity. In addition, sera from mice immunized with yPvCSP-All_FL_ or yPvCSP-All_CT_ in Poly (I:C) were able to recognize the native protein sporozoites isolated from *Anopheles* mosquitoes fed with blood from Thailand volunteers infected with *P. vivax* (Fig. 5).

**Table 1:** Comparative analysis of Ab avidity.

**Antibody**	**Protein**	**% Avidity** [Poly (I:C)]	**% Avidity** [Montanide ISA720]	**% Avidity**
anti-yPvCSP-All_FL_^*^	PvCSP-VK210	42.70 ± 1.04	46.41 ± 6.20	—
	PvCSP-VK247	42.04 ± 8.12	48.29 ± 7.54	
	PvCSP*-P. vivax*-like	53.14 ± 6.76	55.50 ± 7.15	
anti-yPvCSP-All_CT_^*^	PvCSP-VK210	55.89 ± 9.06	49.64 ± 6.72	—
	PvCSP-VK247	58.33 ± 14.17	58.01 ± 14.01	
	PvCSP*-P. vivax*-like	46.22 ± 11.93	48.34 ± 7.94	
mAb anti-VK210	PvCSP-VK210	—	—	81.39 ± 1.57
mAb anti-VK247	PvCSP-VK247	—	—	85.05 ± 2.58

*No significant differences were found between the % avidity analysed (One way ANOVA by Tukey’s post-test).

### Homologous and heterologous prime-boost vaccination protected mice against a PvCSP-transgenic parasite challenge

**Figure 6:**
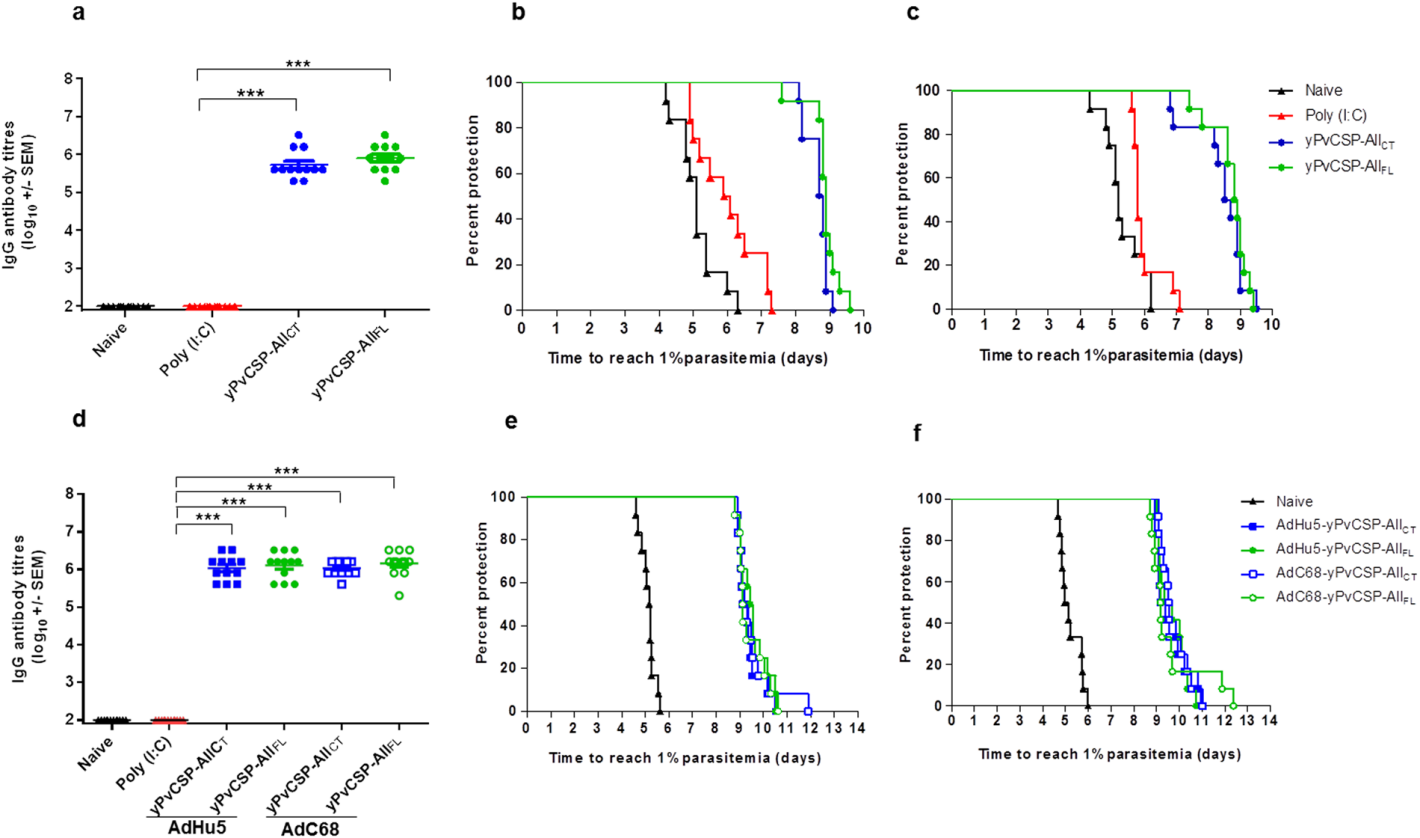
IgG Ab response in immunized mice, and blood-stage parasitemia with the homologous and heterologous prime-boost regimens. In the homologous prime-boost regimen (PPP), groups of 6- to 8-week-old female C57BL/6 mice were immunized subcutaneously (s.c.) with 10 µg of each recombinant protein and Poly (I:C) (50 µg/dose). In the heterologous prime-boost regimen (AdPP), groups of 6- to 8-week-old female BALB/c mice were immunized intramuscularly (i.m.) with AdHu5 or AdC68 at a dose of 10^8^ infection units, and boosted twice with 10 µg of each recombinant protein and Poly (I:C) (50 µg/dose). Twenty-one days after third dose, mice were challenged with 2,000 Pb/PvVK210 spz. or with 2,000 Pb/PvVK247 spz, alternatively. Protection is indicated by the time predicted to reach 1% blood-stage parasitemia using a linear regression model. Blood-stage parasitemia was monitored from day 3 post-challenge until mice reach 1% parasitemia. Titres of IgG Abs against yPvCSP-All_CT_ and yPvCSP-All_FL_ were measured by ELISA. Serum was collected after the third dose. (**a**) and (**d**). IgG Ab titres of the immunized mice using a homologous (PPP) or heterologous prime-boost regimen (AdPP), respectively. (**b)** and (**c**). Time to 1% blood-stage parasitemia post-challenge with Pb/PvVK210 spz (n = 6 per group) or Pb/PvVK247 spz (n = 6 per group) after PPP regimen. (**e**) and (**f**). Time to 1% blood-stage parasitemia post-challenge with Pb/PvVK210 spz (n = 6 per group) or Pb/PvVK247 spz (n = 6 per group) after AdPP regimen. For IgG Ab titre, two independent assays were performed. The results are expressed as the arithmetic mean titres of each group (log_10_ ± SEM) and were compared by one-way ANOVA followed by Tukey’s Multiple Comparison Test. Significant differences between groups are denoted on the graph: ***P < 0.001 and for time to reach 1% of parasitemia, two independent assays were performed. Significant value of ***P < 0.001 was determined by one-way ANOVA with Mantel-Cox test.

**Figure 7:**
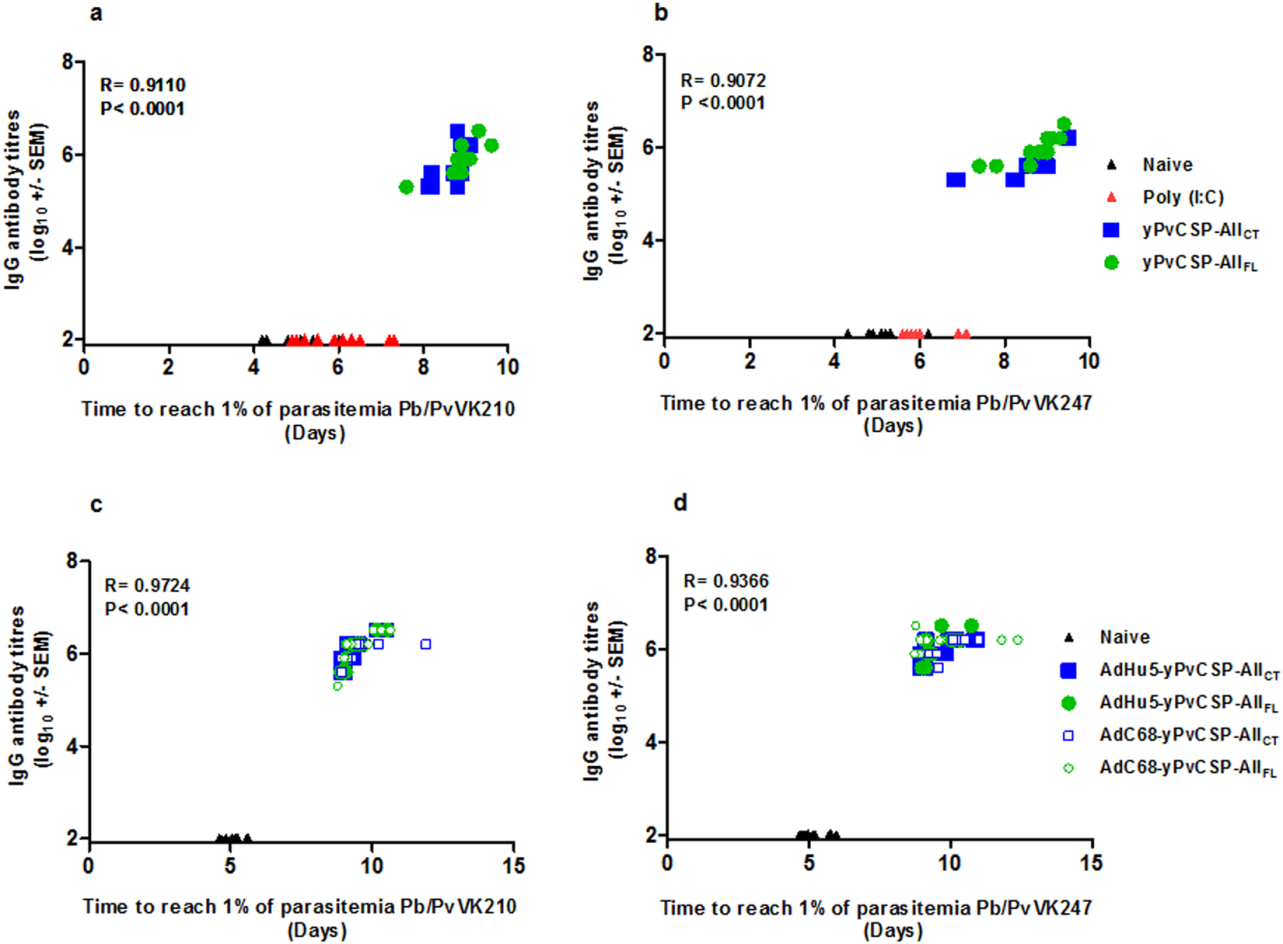
Correlation between titres of IgG Abs pre-challenge against yPvCSP-All and parasitemia post-challenge. Mice were immunized and challenged as described in the legend of Fig. 6. (**a**) and (**b**). C57BL/6 mice were challenged with Pb/PvVK210 spz or Pb/PvVK247 spz, respectively, after PPP regimen. (**c**) and (**d**). BALB/c mice were challenged with Pb/PvVK210 spz or Pb/PvVK247 spz, respectively, after AdPP regimen. Correlation coefficient analyses were determined with a Pearson bivariate, two-tailed test of significance (GraphPad Prism).

In order to analyse the functionality of the anti-PvCS Abs, we used two chimeric parasites (Pb/PvVK210 and Pb/PvVK247) to perform challenge studies in a rodent malaria model^27^. Sera from C57BL/6 mice were highly reactive to the homologous proteins, yPvCSP-All_FL_ or yPvCSP-All_CT_, after the third dose using protein (PPP) in adjuvant Poly(I:C) (Fig. 6a). In experiments to examine protective efficacy of the heterologous prime-boost regimen (AdPP), using prime AdHu5 or AdC68 (Fig. 6d–f), we immunized a second mouse strain (BALB/c). We found that mice immunized using PPP (Fig. 6b,c) or AdPP (Fig. 6e,f) protocols showed a significant delay in the mean pre-patent period (P < 0.001), relative to the control mice injected with Poly(I:C) only. Sterile protection was not induced, however the median time to reach 1% of parasitemia was higher in all vaccinated animals than in the control group, in both vaccination regimens (Table 2). Animals in the control groups reached 1% parasitemia on day 5 after infection, whereas the vaccinated mice presented no parasites in their blood at that time. More relevant, there was a positive correlation between levels of IgG and time to reach 1% of parasitemia in all vaccination schedule (PPP regimen *versus* challenge with Pb/PvVK210, R = 0.911, Fig. 7a; PPP regimen *versus* challenge with Pb/PvVK247, R = 0.907, Fig. 7b; AdPP regimen *versus* challenge with Pb/PvVK210, R = 0.972, Fig. 7c; AdPP *versus* challenge with Pb/PvVK247, R = 0.937, Fig. 7d). A positive correlation was also observed when IgG subclasses were analysed (data not shown).

**Table 2:** Summary of the efficacy induced by the homologous and heterologous vaccination regimens.

Challenge (2,000 spz)	Vaccine Regimen	Mouse Strain	Median Survival (Days)	Mean ± Standard Error (SEM)	Limits
Pb/PvVK210	Naive	C57BL/6	4.89	4.84 ± 0.10	4.22–5.39
	Poly (I:C)		5.03	5.10 ± 0.10^***^	4.73–5.92
	PPP (yPvCSP-All_CT_)		9.17	9.26 ± 0.16^***^	8.20–9.80
	PPP yPvCSP-All_FL_)		9.45	9.36 ± 0.14^***^	8.62–9.92
Pb/PvVK247	Naive	C57BL/6	4.48	4.66 ± 0.13	4.01–5.24
	Poly (I:C)		4.92	4.96 ± 0.16^***^	4.25–5.82
	PPP (yPvCSP-All_CT_)		8.96	9.03 ± 0.12^***^	8.39–9.73
	PPP (yPvCSP-All_FL_)		8.94	9.03 ± 0.18^***^	8.16–10.64
Pb/PvVK210	Naive	BALB/c	5.10	4.96 ± 0.12	4.07–5.56
	AdPP (AdHu5 + yPvCSP-All_CT_)		9.27	9.37 ± 0.14^***^	8.89–10.51
	AdPP (AdHu5 + yPvCSP-All_FL_)		9.48	9.55 ± 0.16^***^	8.88–10.52
	AdPP (AdC68 + yPvCSP-All_CT_)		9.20	9.53 ± 0.24^***^	8.92–11.90
	AdPP (AdC68 + yPvCSP-All_FL_)		9.12	9.44 ± 0.17^***^	8.78–10.63
Pb/PvVK247	Naive	BALB/c	5.05	5.21 ± 0.13	4.67–5.97
	AdPP (AdHu5 + yPvCSP-All_CT_)		9.26	9.62 ± 0.21^***^	8.92–10.95
	AdPP (AdHu5 + yPvCSP-All_FL_)		9.52	9.68 ± 0.18^***^	8.99–10.73
	AdPP (AdC68 + yPvCSP-All_CT_)		9.54	9.73 ± 0.18^***^	9.08–10.27
	AdPP (AdC68 + yPvCSP-All_FL_)		9.17	9.63 ± 0.35^***^	8.72–12.37

Significant differences compared to naïve group are denoted on the table (***P < 0.001 in all cases, One way ANOVA).

## Discussion

Selecting an antigen is one of the crucial steps in developing a vaccine. For a vaccine intended to prevent or delay infection caused by malarial parasites, the antigens exposed on the surfaces of sporozoites and liver-stage parasites are attractive targets that can be used alone or combined with other antigens. The aims of this study were to compare the immunogenicity, avidity, and specific Ab responses in mice of two recombinant proteins based on *P. vivax* CSP and produced in the *P. pastoris* system. Furthermore, the protective efficacy of these vaccine formulations using homologous and heterologous immunization regimens was evaluated in a murine model, in which immunized mice were challenged with transgenic *P. berghei-P. vivax* sporozoites.

The humoral immune response is crucial to block initial infection of the mammalian host by malarial sporozoites^3^. In previous works, we showed that recombinant proteins based on the *P. vivax* CS protein were able to generate high Ab titres in mice, demonstrating their immunogenic potential^23–25^. However, the production and purification processes of such bacterial-produced proteins was a step to be improved; as they were recovered from insoluble fractions and an urea treatment was required to obtain the recombinants. Based on these results, we decided to express recombinant PvCS proteins in *P. pastoris* eukaryotic system, which has been successfully and reproducibly utilised in our previous studies^28,29^. Thus, two fusion-proteins were used in this study: yPvCSP-All_CT_ and yPvCSP-All_FL_. The first construct was formulated based on the CSP portion of the RTS,S vaccine^4^ and is a mix of the three repeat sequences (VK210, VK247, and *P. vivax*-like) with the C-terminal region of PvCSP^30^. As for yPvCSP-All_FL_, this recombinant includes also the PvCSP N-terminal region, since humans immunized with vaccine formulations containing long synthetic peptides representing portions of the N and C terminal of PvCSP generated Abs that inhibited the invasion of sporozoites *in vitro*^31^ and the N-terminal region of *P. falciparum* CSP was proposed to be a target for inducing protection in vaccine formulations^32^.

Our first objective was to produce both proteins in order to compare their immunogenicity in formulations with suitable adjuvants. The recombinants were obtained with a high degree of purity and with proper secondary conformation upon expression in *P. pastoris* system and chromatographic purification. In addition, they were confirmed to be specific for variants PvCSP-VK210 and PvCSP-VK247 by immunoblotting using mAbs. The recombinant antigens induced high titres of IgG Abs specific for the three *P. vivax* CSP variants (VK210, VK247, and *P. vivax*-like) in C57BL/6 mice when administered with either adjuvant Poly (I:C) or Montanide ISA720. High Ab titres using Montanide ISA720 have been reported previously^33,34^ and Poly (I:C) was also demonstrated to be a very potent adjuvant when administered together with malaria antigens^25,35,36^. Thus, immunization with the recombinant proteins described herein was able to generate high titres of Abs that should recognise each natural genetic variant of *P. vivax*. Interestingly, immunization with either yPvCSP-All_CT_ or yPvCSP-All_FL_ emulsified in adjuvant Montanide ISA720 generated IgG Abs reaching a peak in the response after the second dose, whereas in the presence of adjuvant Poly (I:C), a third dose was required to elicit Ab titres of the same magnitude. The precise reason for this difference is not clear, and to our knowledge this is the first report of a comparison between these adjuvants, regarding antibody production.

In spite of these differences after the second dose, there were not significant differences in titres after the third dose. We were interested then in assessing the specificity and avidity of these Abs. The specificity of anti-yPvCSP-All_CT_ and anti-yPvCSP-All_FL_ Abs was evaluated using constructs with only the repeats of each PvCSP variant fused to flagellin (FliC) from *Salmonella enterica* serovar Typhimurium^23,24^. Immunized mice generated high titres of IgG Ab directed against PvCSP repeats, primarily directed at VK210 and *P. vivax*-like variants. Our results are in agreement with those demonstrated for the VMP001 formulation; an effective immune response against *P vivax* should not necessarily be associated with a high number of repeats^13^. High titres of Abs towards the No Repeats protein (N- and C-terminal domains only) were also elicited using both adjuvants. The exact effect of the production of Abs to each PvCSP region on the protection remains unclear so far, and the practical implications of these differences will only be elucidated in a human challenge model. However, if the humoral response plays a major role in protection, such chimeric CSP proteins could generate Abs able to recognise and neutralise sporozoites of all the three allelic variants of *P. vivax*.

The avidity percentage measured in a polyclonal sera indicates the proportion of the antibodies which are able to bind strongly to their antigens; as the chaotropic agent used in this assay disrupts weakly bound antibodies^18^. Although the avidity levels of elicited Abs cannot be considered high in relation to mAbs, the fact of 42–58% of elicited Abs being resistant to the denaturing action of 6 M urea indicates a high avidity of the antigen-Ab bonds of at least 42% of elicited Abs. Moreover, Moon *et al*. (2012) demonstrated that the same avidity level of Ab towards pathogen-mimicking nanoparticle vaccines (VMP001-NPs) was high enough to agglutinate live *P. vivax* sporozoites, indicating their potential as effector antibodies^37^.

The predominant IgG1 Ab response (IgG1/IgG2c ratio >1) found in sera of immunized mice is believed to be related to the stimulation of Th2 cells, which assist in the stimulation of Th1 cells^38^. This predominance of IgG1 was also observed in previous studies by our group, via analysis of sera from mice that were immunized with vaccine formulations containing the recombinant *Pv*CSP fused to FliC^23,24^.

As no statistical differences regarding specificity, avidity or IgG subclasses distribution were observed between antibodies elicited by vaccine formulations containing either adjuvant; we decided to move forward with only one adjuvant. Poly (I:C) has been previously used in several vaccination protocols with PvCSP-based proteins^25^ and the stimulation pathway is already known both in humans and mice. This double-stranded RNA acts by stimulating TLR3 present in antigen-presenting cells that induce signaling through multiple inflammatory pathways, including NF-κB^39,40^. On the contrary, the pathway of stimulation for Montanide ISA720 is not yet known, making it difficult to make a major investment in its use in vaccines. Thus, we opted for the use of Poly (I:C) due to the better knowledge about this adjuvant, both regarding antibody response as well as signaling pathway.

We had previously demonstrated that the heterologous prime-boost regimen, using AdC68-PvCSP or AdHu5-PvCSP for priming and recombinant proteins for boost (AdPP), was as good as the homologous regimen in generating high IgG Ab titres specific for PvCSP antigen^25^. In this study, we confirmed those findings by using yeast-produced instead of bacteria-produced recombinant proteins, and we tested for the first time the protective efficacy of these adenoviruses applied in the AdPP regimen. The reason for using both the Human 5 and the Chimpanzee 68 serotypes (AdHu5-PvCSP and AdC68-PvCSP respectively) in our immunization schedules relies in some concerns related to the use of Human 5 serotype adenovirus as vaccine carriers. Infections with this serotype are frequent in the early life of a significant percentage of the population, and the consequences of the presence of antibodies towards these adenoviruses in pre-immunized subjects are not completely known, both regarding vaccine potency and safety concerns. For these reasons, Simian serotypes as AdC68 are very attractive options^41,42^. However, as the immune response in humans towards AdHu5 is more potent and has been more deeply studied than the AdCh68 serotype, we decided to analyse the protective efficacy of both adenoviruses carrying the PvCSP sequence.

Under our experimental conditions, no significant differences in terms of immunogenicity or protective efficacy were observed among the different formulations and regimens utilized. Since both recombinant proteins under test (yPvCSP-All_FL_ and yPvCSP-All_CT_) combined with suitable adjuvants were able to generate high Ab titers targeting all their different domains in equal proportion, and more important, their protective effect against Pb/Pv sporozoite challenge was also similar, we cannot exclude either of them as vaccine candidates for clinical trials. As for the use of adenovirus for heterologous (AdPP) compared with homologous (PPP) prime-boost regimens, a significant delay in the time to reach 1% parasitemia was seen in 100% of the mice vaccinated with both immunization regimens. Therefore, due to the absence of advantages using the more complex AdPP system, we suggest that the homologous PPP regime would be attractive for clinical trials. It is important to highlight that for malaria sporozoite challenges, a time delay in reaching the liver is an important step in determining protection against liver-stage parasites^16^. A comparable time delay for parasitemia was observed in 59% of individuals vaccinated against *P. vivax* with the vaccine candidate VMP001/AS01B^15^. Even if these vaccination approaches did not induce sterile protection, a significant delay in parasitemia is an important advance, taking into account that a *P. vivax* vaccine with low to moderate effectiveness against infection could hypothetically decrease parasite relapses and thus reduce malaria transmission^43^. In addition, these results would pave the way for approaches to improve strategies for new vaccine formulations.

The results presented in this study support the hypothesis that CSP-based vaccine formulations can induce immunity and protection against the two major PvCSP, VK210 and VK247 alleles. As our proposed formulations cover constructs representing the three allelic variants of PvCSP, they may serve as the basis for a universal vaccine against malaria caused by *P. vivax*.

## Methods

### Gene design and construction of the recombinant proteins

Both CSP-based recombinant proteins used in this study contained immunodominant regions comprising core amino acid repeats from different *P. vivax* alleles (VK210, VK247, and *P. vivax*-like). Although one CSP recombinant contained the N- and C-terminal regions (yPvCSP-All_FL_), another lacked the N-terminal region (yPvCSP-All_CT_). The gene design, construction and expression in *P. pastoris* of yPvCSP-All_CT_ was recently described^30^. As for yPvCSP-All_FL_, a synthetic gene codon-optimised for expression in *P. pastoris* yeast was obtained commercially (GenScript USA Inc., Piscataway, NJ). The chimeric protein presented six copies of the repeat sequence in the classic allele VK210 (GDRA[A/D]GQPA) in their central region, followed by six copies of the repeat in the *P. vivax*-like allele (APGANQEGGAA), and five copies of the repeat in the VK247 allele (ANGAGNQPG). These tandem repeat sequences were followed by the N- and C-terminal portion identical to that in *P. vivax* CSP Salvador I strain sequence. Hexa-histidine (His_6_)-coding sequences were added at the 3′- and 5′-ends of the synthetic CSP gene immediately after and before sites of the restriction enzymes *Eco*RI and *Not*I (New England Biolabs USA Inc., Ipswich, MA) to enable purification. The gene was sub-cloned into vector pPIC9K (Invitrogen, Life Technologies Corporation USA Inc., Waltham, MA), which contains a signal peptide (α-factor) for secreted expression (Invitrogen). Figure 1c,d shows the amino acid sequences of yPvCSP-All_FL_ and yPvCSP-All_CT_ proteins.

### Expression and purification of yPvCSP-All_FL_

Similarly to that described for yPvCSP-All_CT_^30^, the plasmid pPIC9K-*pvcsall*_*FL*_ was linearized with *Sal*I to transform the *P. pastoris* GS115 strain (*his4*^−^) by electroporation following the manufacturer’s recommendations (Invitrogen). Approximately 50 His^+^clones transformed with pPIC9K-*pvcsall*_*FL*_ were screened for high-copy-number integration by G418 selection. Based on an immunoblotting analysis with mouse monoclonal anti-PvCSP-VK210 (2F2) and anti-PvCSP-VK247 (2E10.9)^26^, clones with Mut^+^ phenotype secreting high levels of yPvCSP-All_FL_ were selected. The recombinant proteins were expressed and purified as previously described by our group^28^.

### Characterisation of yPvCSP-All_FL_ and yPvCSP-All_CT_

The recombinant proteins were characterised using a 12% sodium dodecyl sulphate-polyacrylamide electrophoresis (SDS-PAGE) gel stained with Coomassie blue, immunoblotting, reverse-phase chromatography (RP-HPLC), and circular dichroism spectroscopy. Protein expression was confirmed by western blotting. For this analysis, the protein fractions were transferred from a 12% SDS-PAGE gel to a nitrocellulose membrane and exposed to mAbs [anti-His tag, 1:1,000, GE Healthcare; anti-PvCSP-VK210 (2F2), 1:1,000; and anti-PvCSP-VK247 (2E10.E9), 1:1,000 ^26^, followed by anti-mouse horseradish peroxidase (HRP)-conjugated IgG Ab (KPL). The bands were visualised using an ECL Western Blotting Analysis System (GE Healthcare).

The high yield and purity of the recombinant proteins were confirmed by RP-HPLC using a Phenomenex Jupiter C_18_ column (4.6 mm × 250 mm, 5 µm particle, and 300 Å pore) with a Shimadzu Prominence HPLC System Solution (Shimadzu JPN Corp., Kyoto, KY). The HPLC procedure was performed using a 90% acetonitrile gradient in 0.1% trifluoroacetic acid at room temperature (RT, 22 °C) at a flow rate 1 mL/min for 30 min. A UV-visible absorbance detector (DAD: diode array detector, Shimadzu SPD M20A) was used for detection at 214 nm as described^44^.

The secondary structure of the recombinant proteins was analysed by circular dichroism (CD), using a JASCO-J815 spectropolarimeter (Jasco, Tokyo, Japan) at 25 °C. The purified proteins (diluted to 10 µM in phosphate buffered saline (PBS)) were loaded into a 5-mm quartz cuvette. Far-UV measurements (four scans) were obtained at wavelengths of 250–190 nm at 0.1-nm intervals, with a 1-nm bandwidth and 1 s response time. The spectrum presented is the average of the four scans, and the data obtained are reported as values of molar ellipticity [θ]MRW (deg × cm^2^ × dmol^-1^). A baseline measurement with PBS was subtracted from each protein spectrum. The data were smoothed with a Savitzky-Golay filter, and the percentages of secondary structure were predicted using the CD Analysis and Plotting Tool (http://capito.nmr.leibniz-fli.de/index.php).

### Recombinant proteins expressed in *E. coli*

Previously, we described the generation of three recombinant proteins representing each PvCSP variant (VK210, VK247, and *P. vivax*-like; constructs with the N- and C- terminal portions^25^), FliC-MSP1_19_-PADRE (non-related protein containing FliC^45^), and the three FliC-PvCS-repeats (FliC-PvCSP-VK210, FliC-PvCSP-VK247, and FliC-PvCSP-*P. vivax*-like; constructs without the N- and C-terminal portions^23,24^).

### Adenoviral vectored vaccines

Recombinant replication-defective human adenovirus serotype 5 (AdHu5) and chimpanzee adenovirus serotype (AdC68) vectors with the sequence Ad-PvCSP (containing the three-repeat variants plus N- and C-terminal regions in fusion) were generated previously^25^. These adenoviruses were amplified in HEK 293 cells; purification, quality control, and quantification were performed as described^46^. The adenoviruses were used in the heterologous prime-boost vaccination regimen.

### Mouse immunizations

#### Homologous prime-boost regimen (PPP) in two adjuvants for comparative studies

Groups of six female C57BL/6 (H-2^b^) inbred mice (n = 6/group) aged 6–8 weeks (Harlan, UK) were used in all experiments. Immunizations were performed by subcutaneous (s.c.) administration of 10 µg of the chimeric protein in presence of the adjuvant Poly (I:C) (HMW, 50 µg/ dose; Invivogen) or Montanide ISA720 (7:3), SEPPIC], three times at 21-day intervals. The controls received only PBS emulsified in the adjuvant. Twenty-one days after each immunization, blood was collected from tails, and sera were stored at −20 °C until use.

#### Homologous (PPP) and heterologous (AdPP) prime-boost regimens in an experimental challenge

Mice were exposed to the homologous prime-boost vaccination regimen (PPP) after anesthetising using an inhalation chamber containing a mix of isoflurane (23.5%) and oxygen (12 L/min). Mice were immunized (s.c.) three times at 2-week intervals, with a 100 μL dose containing sterile PBS and 10 μg/dose of recombinant protein (yPvCSP-All_CT_ or yPvCSP-All_FL_) with 50 μg/dose of Poly (I:C) HMW adjuvant (Invivogen). One control group received only PBS with 50 μg/dose of Poly (I:C). A naïve group was also used as a control.

For the other arm, the AdPP vaccination regimen was administered to female BALB/c (H-2^d^) mice 6–8 weeks of age purchased from Harlan/UK. Mice were immunized three times at 2-week intervals. Mice were primed with AdHu5 or AdC68 at a dose of 10^8^ infectious units (IU), administered intramuscularly (i.m.) into the tibialis anterior, with 25 μL in each leg, for a final volume of 50 μL per mouse. Later, the mice were boosted twice using the recombinant protein formulations described above. Fifteen days after each immunization, blood was collected from tails, and sera were stored at −20 °C until use.

#### Detection of Ab titres to each PvCSP variant

Collected sera were analysed for the presence of Abs against the recombinant proteins described above. Abs to the PvCSP variants (VK210, VK247, and *P. vivax*-like), FliC-MSP1_19_-PADRE (non-related protein containing FliC), and the three FliC-PvCSP-repeats in mice sera were detected by ELISA on days 21, 42, and 63, as previously described^45^. The same protocol was used to detect Abs against yPvCSP-All_FL_ and yPvCSP-All_CT_ in sera from mice exposed to homologous and heterologous prime boost-regimens on days 15, 30, and 45 after priming and 4 days after challenge. The specific titres were considered the highest dilution yielding an OD_492_ greater than 0.1. The results were expressed as means of IgG titres (log_10_)/SEM. The avidity of IgG Abs was determined using an extra step of incubation with 6 M urea for 5 min. The percentage was defined as avidity (OD_492/630_ with treatment)/(OD_492_/_630_ untreated) × 100%^18,37^. As controls, mAb anti-VK210 [1: 500]) and anti-VK247 [1: 500] were used.

#### *P. vivax* slide preparation and indirect immunofluorescence assay

*Anopheles cracens* mosquitoes were fed on human blood obtained from infected Thai patients using a membrane-based feeder system^47^. Blood sample collection from malaria patients was performed in accordance with the relevant guidelines and regulations and was approved by Oxford Tropical Research Ethics Committee (Reference 28-09). After two weeks, aseptically dissected infected salivary glands were disrupted in a glass tissue grinder and the sporozoite preparation was deposited on glass slides. Slides were dried at room temperature and kept frozen at -20C before use. The glass slides were fixed with metanol for 10 min and blocked with BSA 5% in PBS for 30 min. Sera from C57BL/6 mice immunized with yPvCSP-All_FL_ or yPvCSP-All_CT_ in the presence Poly (I:C) (dilution 1:50) were applied to the slides and incubated for 1 h. The slides were then washed 3 times with PBS before the addition of anti-mouse IgG conjugated to Alexa Fluor 568 (Molecular Probes) diluted 1:500 with 5% BSA in PBS. After washed 3 times with PBS the cells were stained with DAPI (2 μg/mL). The images were acquired on a DMI6000B/AF6000 (Leica Systems) fluorescence microscope coupled to a digital camera system (DFC 365 FX). As a positive control, was used the monoclonal antibody anti-VK210 (1:50).

#### Parasite preparation and experimental challenge

*P. berghei* ANKA transgenic parasite line 2196cl01 expressing PvCSP-VK210 (Pb/PvVK210) and line 2199cl1 VK247 (Pb/PvVK247) sporozoites (spz) used for challenge were produced at the Jenner Institute insectary, Oxford University^27^. Donor mice were infected by intraperitoneal (i.p.) injection of cryopreserved infected blood stocks. Exflagellation was first confirmed and *Anopheles stephensi* mosquitoes were exposed to anesthetised infected donor mice for 10 min. After 21 days in a humidified incubator at 19–21 °C and on a 12-h day-night cycle and fed fructose–p-aminobenzoic acid (PABA) solution, the mosquitoes were hand-dissected, salivary glands were isolated, and sporozoites were extracted and resuspended in Schneider’s media (Pan Biotech). The total number of sporozoites was determined using a haemocytometer. Experimental challenges were performed intravenously using 2,000 freshly isolated sporozoites at day 21 after the last immunization.

#### Thin blood smears to assess parasitemia

Thin blood smears were prepared daily, from day 3 after infection until the mice reached 1% parasitemia. The smears were prepared on glass slides with a drop of blood obtained from a tail clip. The blood smear was allowed to air-dry before fixation with methanol and staining for at least 15 minutes using 10% Giemsa diluted in distilled H_2_O. The slides were then allowed to air dry at room temperature. Using a light microscope, parasitemia was quantified as the percentage of infected erythrocytes in at least 1,000 RBCs per mouse. To determine the time required to reach 1% blood-stage parasitemia, a linear regression model was used, as previously described^16^. The time to reach 1% parasitemia was used in survival analyses to assess vaccine efficacy.

#### Statistical analysis

All analyses were performed using GraphPad Prism version 5.0 (GraphPad Software Inc., La Jolla, CA, USA) and graphics were plotted using SigmaPlot version 11.0 (GraphPad Software, La Jolla, CA, USA). A comparison of Ab titers in independent samples was performed by one-way analysis of variance (ANOVA). One-way ANOVA was also used to compare normally distributed log-transformed means for the different animal groups. Multiple comparisons were assessed by Tukey’s post-test with a significance level of P < 0.05. The significance in survival curves was determined using a log-rank Mantel-Cox test with a significance level of P < 0.05. Correlation coefficient analyses were determined with Pearson bivariate, two-tailed test of significance.

#### Ethics statement

This study was performed in strict accordance with the recommendations of the Guide for the Care and Use of Laboratory Animals of the Brazilian National Council of Animal Experimentation (http://www.cobea.org.br/). The protocol (CEUA/FCF no. 475/2014) was approved by the Research Committee on Animal Experimentation of the School of Pharmaceutical Sciences of the University of São Paulo, Brazil. All animals and procedures for the homologous and heterologous assays performed at the Jenner Institute were conducted in accordance with the terms of the UK Home Office Animals Act Project License. Procedures were approved by the University of Oxford Animal Care and Ethical Review Committee (PPL30/2889).

The blood samples were collected from malaria patients infected by *P. vivax* (Mae Sot region, Thailand). All patients here used signed an informed consent form after receiving detailed information regarding the study’s goals. The study was approved by Oxford Tropical Research Ethics Committee (reference OX28-09).
